# Imaging and pathological features of primary hepatic neuroendocrine carcinoma: An analysis of nine cases and review of the literature

**DOI:** 10.3892/ol.2014.1844

**Published:** 2014-01-31

**Authors:** ZHU CHEN, HUA-EN XIAO, PAUDEL RAMCHANDRA, HAI-JIANG HUANG

**Affiliations:** 1 Department of Radiology, The Second Xiangya Hospital of Central South University, Changsha, Hunan 410011, P.R. China; 2 Department of Pathology, The Second Xiangya Hospital of Central South University, Changsha, Hunan 410011, P.R. China

**Keywords:** primary hepatic neuroendocrine carcinoma, computed tomography, magnetic resonance imaging, pathology

## Abstract

The present study aimed to analyze the imaging features and pathological basis of primary hepatic neuroendocrine carcinoma (PHNEC). A retrospective analysis of the imaging and pathological features of nine PHNEC cases was carried out at The Second Xiangya Hospital of Central South University (Changsha, China). The nine patients were subjected to dynamic contrast-enhanced computed tomography (CT) scanning of the liver and pathological diagnosis of the tissue samples. In addition, two patients were subjected to magnetic resonance imaging (MRI). CT scanning revealed the presence of single or multiple masses in the liver with a maximum diameter of 1–10 cm. These hepatic masses were of low density as showed by plain CT. These masses showed uneven or annular enhancement at their margins in the arterial phase. The venous portal phase showed consistent or declined enhancement and the delayed phase showed light enhancement in these masses. In addition, multiple intrahepatic nodules with long T1 and T2 signal intensities and obvious enhancement were observed by MRI in one patient, while intrahepatic lesions with moderate length T2 signal intensities and light enhancement not visible on the T1 image were observed in another patient. Pathological analysis revealed that the tumor cells exhibited morphological diversity. Immunohistochemical staining revealed that the tumor cells were chromogranin A- and synaptophysin-positive, and carcinoembryonic antigen-, hepatocytic antigen- and α-fetoprotein-negative. Imaging methods, including CT and MRI, are useful for the diagnosis of PHNEC; however, pathological examination is required for a final, definite diagnosis.

## Introduction

Neuroendocrine tumors (NETs) are a rare type of tumor, originating from dispersed neuroendocrine cells distributed throughout the body. These cells have the ability to synthesise, store and secrete neurohormones, neurotransmitters and neuromodulators and can be either functioning or non-functioning. Functioning NETs may lead to carcinoid syndrome due to the secretion of serotonin and other vasoactive hormones including gastrin, insulin, glucagon and somatostatin. The symptoms of NETs may lead to misdiagnosis as may manifest in the abdominal organs in addition to the neuroendocrine system, thereby complicating patient treatment. Therefore researchers are attempting to diagnose this tumor type early in order to commence treatment sooner. This tumor type predominantly affects the gastrointestinal tract and pancreas rather than the liver. Primary hepatic neuroendocrine carcinomas (PHNEC) are a rare tumor type which are reported to have an incidence of 1.5/100,000 (1), accounting for <1% of all malignancies (2). Alternatively, due to carcinoid syndrome the symptoms of PHNEC may be varied and different to those for primary hepatocellular carcinoma (HCC) or those of other liver tumors. This feature and the rarity of PHNEC make it difficult to diagnose the PHNEC early and accurately prior to performing biopsies or tumor resectioning. To date, PHNEC is often misdiagnosed prior to performing a pathological examination. Computed tomography (CT) and magnetic resonance imaging (MRI) are emerging as two clinically usefully techniques in oncology. These techniques may be used for diagnosis, treatment monitoring and pathophysiologic understanding of PHNEC. However, it is often impossible to diagnose PHNEC accurately by relying solely on CT or MRI and pathological examination is currently the gold standard for diagnosis. To improve knowledge and diagnosis of PHNEC, the present study analyzed the imaging results and pathological features of nine pathologically diagnosed cases.

## Materials and methods

### Clinical materials

Nine cases of PHNEC diagnosed by pathological examination following surgery and biopsy between 2007 and 2012 were collected from the Second Xiangya Hospital of Central South University (Changsha, China). Patients included six males and three females aged between 24 and 66 years with an average age of 46.2 years. Tumor marker serum analysis showed the nine patients to be α-fetoprotein (AFP)-negative. Samples were also cancer antigen 125 (CA125)-, carbohydrate antigen 19-9 (CA19-9)-, carcinoembryonic antigen (CEA)- and neuron-specific enolase (NSE)-negative in six cases. One patient was HBsAg-positive. Six patients exhibited symptoms of abdominal pain and distension, two had alimentary tract bleeding, two had anorexia and exhibited weight loss and one patient had jaundice. This study was approved by the Ethics Commitee of The Second Xiangya Hospital of Central South University (Changsha, China). All patients provided written informed consent.

### Equipment and methods

The nine patients were subjected to computed tomography (CT) scanning. Eight were subjected to 64-slice spiral CT (Siemens Corporation, Munich, Germany). Patients were tested by plain and dynamic contrast-enhanced scanning of the liver with a matrix of 512×512, layer depth of 5 mm, layer distance of 5 mm and a vision field of 25×25 to 38×38 cm, using iohexol (dose, 1.5 ml/kg; speed of injection, 2.5 ml/sec) as a contrast medium. Arterial, portal venous and delayed phase scans were performed at 25–30 sec, 50 sec and 5 min respectively for all phases, following injection of the patients with contrast medium. The other patient was subjected to LightSpeed 16 Slice CT (GE Healthcare, Little Chalfont, UK). The patient was tested by dynamic CT scanning of the liver with a matrix of 512×512, layer depth of 5 mm, layer distance of 5 mm and a vision field of 50×50 cm, using iohexol as a contrast medium (dose, 80 ml; speed of injection,3.5 ml/sec).

Two patients were subjected to magnetic resonance imaging (MRI) testing using the 1.5T Signal Twin Speed MRI scanner (GE Healthcare). The parameters for T1-weighted image (T1WI) were listed as follows: Echo time (TE), minimum 1.6 msec; repetition time (TR), 150 msec; matrix, 256×160; number of excitations (NEX), 2.00; field of view (FOV), 36×36 cm; layer depth, 5 mm; layer distance, 1.5 mm; and fast spoiled gradient*-*recalled echo-sequence cross-section scanning during a respiratory cycle. The parameters for T2-weighted image (T2WI) were as follows: TE, 102 msec; TR, 4,000 msec; matrix, 256×256; NEX, 4.00; FOV, 36×36 cm; layer depth, 5 mm; layer distance, 1.5 mm; and fat suppression fast recovery fast spin echo sequence with respiratory triggering technique transverse and cross-section scanning. The two patients were injected with gadolinium diethylene-triamine pentaacetic acid as contrast medium at a dose of 0.1 ml/kg. The parameters used for enhancement scanning were: Axial FAME ASSET sequence; TE in phase; TR, 20 msec; FOV, 38×38 cm; NEX, 1.0; layer depth, 4 mm; layer distance, 0 mm; and matrix 256×160. This was performed using transverse scanning.

### Pathology techniques

Four patients were subjected to surgical biopsy, while the remaining five patients were subjected to CT-guided puncture biopsy. Samples were cut into slices and stained with hematoxylin-eosin and other immunohistochemical staining agents, including chromogranin A (CgA), synaptophysin (Syn), NSE, cytokeratin (CK), hepatocytic antigen (HPC), CEA, AFP, epithelial membrane antigen (EMA) and vimentin (Vim) (Maxin-Bio, Fuzhou, China).

## Results

### Imaging results

#### CT results

CT scanning revealed single or multiple masses in the livers of the patients, with a maximum diameter of 1–10 cm. These hepatic masses were shown by plain CT to be of low density. The single tumor masses exhibited clear boundaries, whereas the multiple masses exhibited unclear boundaries and an uneven density. A liquefied necrotic area of lower density was observed in the center of certain larger lesions. Furthermore, the arterial phase of dynamic enhancement CT showed uneven or annular enhancement of the tumor margins. The venous portal phase showed consistent or declined enhancement in the tumor masses, whilst the delayed phase showed light enhancement.

**Figure 1:**
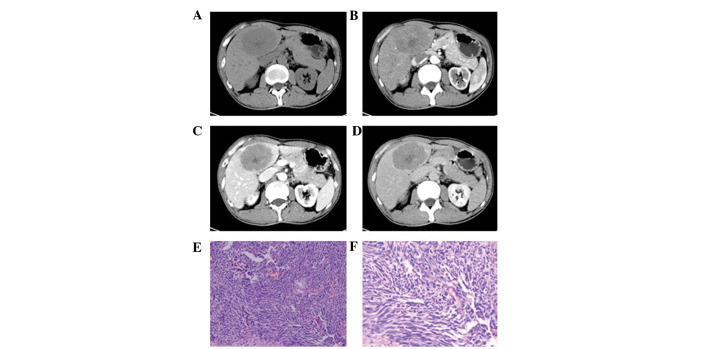
Poorly differentiated neuroendocrine carcinoma in a 57-year-old male. (A) Precontrast computed tomography showed a single mass in the left hepatic lobe with a maximum cross-section size of 6.5×5.0 cm, clear boundaries and uneven density. A liquefied necrotic area in the center of the focus was observed. (B) In enhancement scanning, a mild and uneven enhancement was observed at the edge of the mass in the arterial phase. (C and D) A decline in enhancement was observed in the portal venous and delayed phases, respectively. (E and F) Hematoxylin-eosin staining (magnification, ×100 and 200, respectively) showed the poorly differentiated cancer cells to be smaller, with less cytoplasm, angular and trachychromatic nuclei and karyokinesis.

One patient exhibited a single mass in the left hepatic lobe with a maximum cross-section size of 6.5×5.0 cm, clear boundaries and an uneven density (Fig. 1). A liquefied necrotic area in the center of the focus was observed. Enhancement scanning revealed a mild and uneven enhancement at the edge of the mass in the arterial phase, which declined in the portal venous and delayed phases.

**Figure 2:**
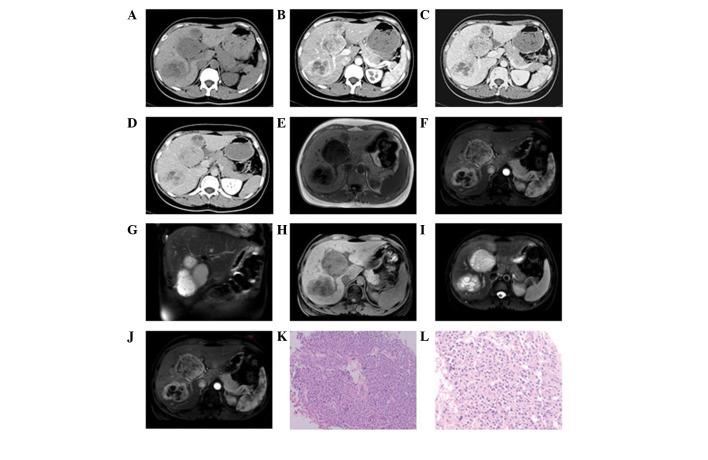
Well-differentiated neuroendocrine carcinoma in a 34-year-old female. (A) Precontrast computed tomography showed multiple lumps of various sizes in the right and left hepatic lobes with a maximum cross-section size of 6×5 cm, clear boundaries and uneven density. A liquefied necrotic area in the center of the largest focus was observed. (B) In enhancement scanning, an annular enhancement was observed in the arterial phase. (C and D) A decline in the enhancement was observed in the portal venous and delayed phases, respectively. (E–J) Magnetic resonance imaging showed multiple long T1 and T2 signal foci in the liver, which were nodular and lumpy which were markedly enhanced. (K and L) Hematoxylin-eosin staining (magnification, ×100 and 200, respectively) revealed morphological diversity of the tumor cells and vessel-like arrangement in parts, with similar morphological features and limited interstitial substance.

**Figure 3:**
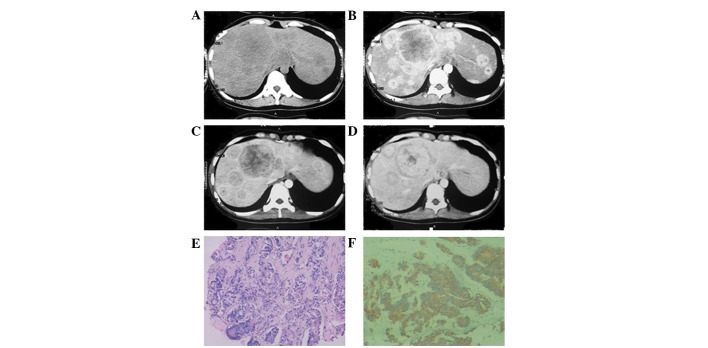
Carcinoid in a 24-year-old female. (A) Precontrast computed tomography showed multiple lumps of various sizes in the right and left hepatic lobes with obscure boundaries, uniform density and a maximum diameter of 7.5 cm. (B) In enhancement scanning, obvious enhancement was observed in the arterial phase and an annular enhancement was shown in the largest mass. (C and D) A decline in enhancement was observed in the portal venous and delayed phases, respectively. (E and F) Hematoxylin-eosin staining (magnification, ×100) and immunohistochemical staining, respectively. The tumor cells showed morphological diversity and vessel-like arrangement in parts, with similar morphological features and limited interstitial substance.

**Figure 4:**
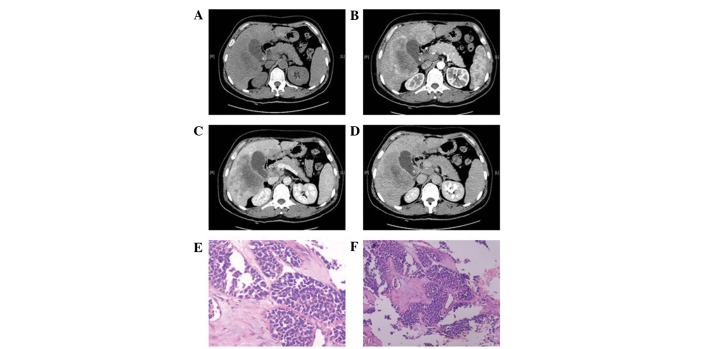
Poorly differentiated neuroendocrine carcinoma in a 46-year-old female. (A) Precontrast computed tomography showed multiple lumps of various sizes in the right and left hepatic lobes with obscure boundaries, uniform density and a maximum diameter of 10 cm. (B) In enhancement scanning, obvious enhancement was observed in the arterial phase and an annular enhancement was identified in the largest mass. (C and D) The enhancement was observed to decline in the portal venous and delayed phases, respectively. (E and F) Hematoxylin-eosin staining (magnification, ×200 and 100, respectively) revealed the poorly differentiated cancer cells to be smaller, with less cytoplasm, angular and trachychromatic nuclei and karyokinesis.

**Figure 5:**
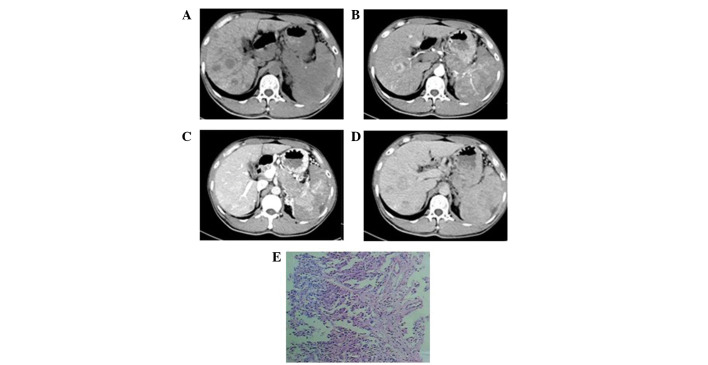
Well-differentiated neuroendocrine carcinoma in a 43-year-old female. (A) Precontrast computed tomography showed multiple intrahepatic lumps with clear boundaries, uniform density and diameters of <2cm. (B) In enhancement scanning, an annular enhancement was observed in the arterial phase and (C and D) the enhancement was seen to decline in the portal venous and delayed phases, respectively. (E) Hematoxylin-eosin staining (magnification, ×100) revealed morphological diversity of the tumor cells and vessel-like arrangement in parts, with similar morphological features and limited interstitial substance.

**Figure 6:**
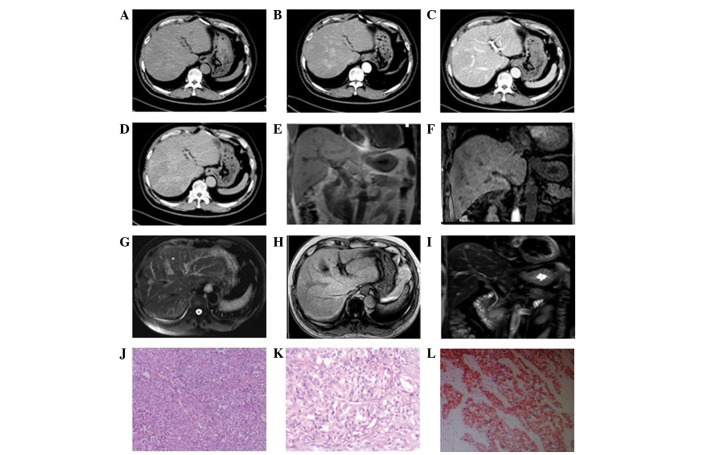
Poorly differentiated neuroendocrine carcinoma in a 57-year-old male. Foci were (A) not visible in plain computed tomography, (B) were clearly visible in the arterial phase and (C) disappeared in the portal venous and (D) delayed phases. No multiple intrahepatic nodules were observed in (E) magnetic resonance imaging T1- and (F) T2-weighted images. Relatively long signals were observed in (G) T2-weighted fat suppression and (H) T1 enhancement images. (H) The foci were unevenly enhanced in T1 enhancement scanning. (J and K) HE staining (magnification, ×100 and 200, respectively) and (L) immunohistochemical staining revealed a number of tumor cells to be uniformly small- to medium in size, with unclear cytoplasmic boundaries and round and regular nuclei arranged flakily, uniformly and in clusters, or like a chrysanthemum.

In the eight patients exhibiting multiple intrahepatic lumps, the foci were of various sizes. In four patients, the diameters of the largest foci were >7 cm whilst in the remaining four, the diameters were <2 cm. Plain CT scan showed low-density lesions. The four cases with larger masses had a lower density liquefied necrotic area (Figs. 2A, 3A and 4A) while the four cases with smaller foci had a relatively uniform density (Fig. 5A). The boundaries of the foci were unclear in six patients (Figs. 3 and 5), while they were clear in the other two cases (Fig. 2). All foci showed uneven enhancement in the arterial phase, six of which exhibited annular enhancement (Figs. 2B–4B). In six patients, the degree of enhancement was found to decline in the portal venous phase (Figs. 2C, 3C and 4C) and the delayed phase (Figs. 2D, 3D and 4D). In two patients, the enhanced area was enlarged in the portal venous phase (Fig. 5C) and the enhancement extended over a long period of time. The degree of enhancement in the delayed phase (Fig. 5D) declined in all cases, and the density of the foci was generally uniform. In one patient (Fig. 6), the foci were not observed in plain scanning but were clearly observed in the arterial phase and absent in the portal venous and delayed phases.

In this group, CT scanning was able to predict the pathological changes and, therefore, the malignant changes. However, a definitive diagnosis depends on pathological examination.

#### MRI results

Two patients were subjected to MRI. In one case (Fig. 2E–J), multiple long T1 and T2 signal foci were observed in the liver, which were nodular, lumpy and significantly enhanced. Another patient (Fig. 6E–I) exhibited multiple intrahepatic nodules that were not clearly visible in T1- or T2-weighted images. However, a relatively long signal was observed in T2-weighted fat suppression and T1 enhancement images. In addition, the foci were unevenly enhanced in T1 enhancement scanning.

#### Pathology

A number of different types of neuroendocrine tumor were observed in this study, including carcinoid tumors, a well-differentiated neuroendocrine carcinoma and a poorly differentiated neuroendocrine carcinoma. Among these were two cases of carcinoid tumors (Fig. 3E and 3F), three cases of well-differentiated neuroendocrine carcinomas (Figs. 2K and 5E) and four cases of poorly-differentiated neuroendocrine carcinomas (Figs. 1F, 4E and 6J–L). Pathological results showed that the tumor cells were morphologically diverse and that a number of tumor cells formed vessel-like arrangements, with similar morphological features and of little interstitial substance (Figs. 2K, 3E and 3F). Other tumor cells were uniformly small- to medium in size with unclear cytoplasmic boundaries. Additionally, their nuclei were round or regular in shape and arranged irregularly, clustered and flakily or like a chrysanthemum (Fig. 6J–L). The poorly differentiated cancer cells were smaller and with less cytoplasm than the well-differentiated cells. Their nuclei were angular, trachychromatic and karyokinesis was observed (Figs. 1F and 4E). In addition, neuroendocrine granules were observed by electron microscopy (H-7500 Electron Microscope, Hitachi, Tokyo, Japan). The immunohistochemical staining in these tumor cells was positive for CgA and Syn and negative for CEA, HPC and AFP (Table I).

## Discussion

Neuroendocrine carcinomas most commonly develop in the gastrointestinal tract and pancreas. Those seen in the liver are metastasized from primary carcinomas of the gastrointestinal tract or pancreas in the majority of cases, and primary neuroendocrine carcinomas originating from the liver itself are rare (3). Symptoms and signs are not obvious in the early stages of this disease type. A number of patients may exhibit nonspecific symptoms, including abdominal pain and distension, whilst several suffer from carcinoid syndrome (4). The condition worsens with progression of the disease, with a number of manifestations, including symptoms caused by tumors compressed in adjacent organs, dyspepsia, weight loss and fatigue. Patients usually deny histories of hepatitis or cirrhosis (5) and exhibit AFP-negative serum levels. In addition, conventional tumor markers, for example CEA, CA125 and CA19-9, are usually negative.

Neuroendocrine tumors originating from neuroendocrine cells may be divided into two types, neurological and epithelial (6). Hepatic neuroendocrine tumors fall into the epithelial subtype but there are conflicting opinions, as it has been hypothesized that hepatic neuroendocrine tumors are formed by proliferation of neuroendocrine cells dispersed within the hepatic biliary epithelium (7). According to the latest World Health Organization criteria (2010) (8), neuroendocrine carcinomas may be subcategorized into three groups: Well-differentiated neuroendocrine tumors (carcinoid), moderately differentiated neuroendocrine carcinomas (atypical carcinoid) and poorly differentiated neuroendocrine carcinomas (small cell neuroendocrine carcinoma).

Pathological results, particularly immunohistochemical results, are required for the definitive diagnosis of a neuroendocrine carcinoma. It is generally accepted that positive expression of CgA, Syn and NSE represents definite evidence for diagnosis (9–11). Among the immunohistochemical results, CgA and Syn staining were positive in the nine patients, which was consistent with the aforementioned studies. NSE testing was performed in three patients, all of which were positive. All nine patients demonstrated negative results for CEA, HPC and AFP, whereas the majority of CK results were positive. Irregular data were obtained in Vim and EMA tests. However, in contrast to the immunohistochemical results, serum NSE tests were negative in all six patients tested, which indicated that NSE was expressed in tumor cells but not in serum. Therefore, this mechanism must be investigated further.

It is difficult to distinguish primary hepatic neuroendocrine carcinomas from metastases using pathological evidence alone. Therefore, clinical features are also important for this clarification. Thorough testing prior to surgery, examination during surgery and follow-ups after surgery are important in determining whether a primary focus is present outside the liver (12).

Neuroendocrine carcinomas are receiving considerable attention and pathological examination techniques, particularly the applications of electron microscopy and immunohistochemistry, are being developed rapidly. Although imaging methods, including CT and MRI, are unable to definitively diagnose neuroendocrine carcinomas (13,14), they remain useful for the preliminary diagnosis of a tumor. This is particularly true when combined with clinical features and other supporting information, for example no patient history of hepatic cirrhosis and an absence of tumor markers, i.e., AFP (3).

The patients of the present study exhibited single and multiple low-density masses with undefined boundaries, the majority of which had uniform density. Liquefaction necrosis was observed in the center of specific larger lesions. Contrast-enhanced CT scanning showed annular enhancement or small-sized heterogeneous enhancement in the arterial phase. The area of enhancement enlarged in the portal venous phase and the density remained almost constant in this phase. There was mild enhancement in the delayed phase. These results are consistent with previous observations (15,16).

In the present study, one patient exhibited lesions only in the left lobe of the liver, while the other eight exhibited multiple lesions in the whole liver, indicating diffuse growth of the tumor. In addition, the majority of the tumors showed annular enhancement. Two patients showed enlargement of the hepatic portal lymph nodes; one showed abdominal lymph node enlargement and the other showed retroperitoneal lymph node enlargement. Portal vein tumor thrombi and cirrhosis were not observed in any of the patients. CT scanning was unable to conclusively diagnose hepatic neuroendocrine carcinomas due to its non-specificity. However, it was able to clearly reveal the internal structure, blood supply, association with neighboring organs and metastasis of the tumor. This indicates that CT may improve preliminary differential diagnosis and determine whether a mass is primary or secondary, the correct treatment and the prognosis.

Two of the nine patients were examined by MRI and showed marginally different appearances. One showed low signal lesions at T1WI whilst the other did not show any abnormal signal in the same MRI sequence. The two patients showed high signal lesions at T2WI, consistent with previous studies (17–20).

This neuroendocrine carcinoma tumor type should be distinguished from primary hepatocellular carcinoma (HCC) irrespective of whether single or multiple in number. A typical HCC often accompanies hepatitis and hepatic cirrhosis, and the majority of patients have elevated serum AFP levels. In addition, HCCs show well-defined enhancement in the arterial phase and rapid loss of enhancement during the portal venous and delayed phases known as the ‘quick in and out of contrast medium’. These factors are important for differential diagnosis. However, specific neuroendocrine carcinomas, with diffuse lesions, are difficult to distinguish from diffuse HCCs. Multiple lesions with ring-shaped enhancements are commonly found, which should be distinguished from metastatic tumors by clinical and immunohistochemical analyses. In addition, HCC should be distinguished from focal nodular hyperplasia (FNH). FNH has a lower degree of enhancement and approximately one-third of FNH patients exhibit a central fibrous scar in lesions, showing a star-shaped low-density shadow in plain and enhanced scanning.

Early detection and resection of primary lesions is the preferred treatment for the improvement of survival rate. For patients without indications for surgery, hepatic artery chemotherapy embolism is a preferred non-operative treatment that results in a good prognosis (21). Radiofrequency ablation, cryotherapy, microwaving and anhydrous alcohol injection also exert therapeutic effects (3,22). Liver transplantation is considered in special cases (23,24). Previous studies have indicated that interferon and somatostatin, as well as its analogs, may be used in the treatment of carcinomas (25,26).

As with other malignant tumors, early detection and treatment are the key to achieving a good prognosis. The prognosis is associated with a number aspects, including pathological type, degree of differentiation, size and boundary of the tumor, metastasis and the physical status of the patient.

## Figures and Tables

**Table I:** Immunohistochemistry results of the nine patients.

Patient no.	CgA	Syn	CK	CEA	HPC	NSE	AFP	Vim
1	++	++	+	−	−		−	
2	+	+	−	−				−
3	++	+			−			−
4	++	++	+	−	−		−	−
5	+	+		−		+	−	+
6	+	+	+		−	+		
7	+	+			−		−	
8	+	+	++	+			−	
9	++	++	−	−		+	−	−

CgA, chromogranin A; Syn, synaptophysin; CK, cytokeratin; CEA, carcinoembryonic antigen; HPC, hepatocytic antigen; NSE, neuron-specific enolase; EMA, epithelial membrane antigen; AFP, α-fetoprotein;Vim, vimentin.
